# Daily milk yield correction factors: What are they?

**DOI:** 10.3168/jdsc.2022-0230

**Published:** 2022-12-01

**Authors:** Xiao-Lin Wu, George R. Wiggans, H. Duane Norman, Asha M. Miles, Curtis P. Van Tassell, Ransom L. Baldwin VI, Javier Burchard, Joao Durr

**Affiliations:** 1 Council on Dairy Cattle Breeding, Bowie, MD 20716; 2 Department of Animal and Dairy Sciences, University of Wisconsin, Madison 53706; 3 USDA, Agricultural Research Service, Animal Genomics and Improvement Laboratory, Beltsville, MD 20705-2350

## Abstract

Cows are typically milked 2 or more times on a test-day, but not all these milkings are sampled and weighed. The initial approach estimated a test-day yield with doubled morning (AM) or evening (PM) yield in the AM-PM milking plans, assuming equal AM and PM milking intervals. However, AM and PM milking intervals can vary, and milk secretion rates may be different between day and night. Statistical methods have been proposed to estimate daily yields in dairy cows, focusing on various yield correction factors in 2 broad categories: additive correction factors (ACF) and multiplicative correction factors (MCF). The ACF are evaluated by the average differences between AM and PM milk yield for various milking interval classes, coupled with other categorical variables. We show that an ACF model is equivalent to a regression model of daily yield on categorical regressor variables, and a continuous variable for AM or PM yield with a fixed regression coefficient of 2.0. Similarly, a linear regression model can be implemented as an ACF model with the regression coefficient for AM or PM yield estimated from the data. The linear regression models improved the accuracy of the estimates compared with the ACF models. The MCF are ratios of daily yield to yield from single milkings, but their statistical interpretations vary. Overall, MCF were more accurate for estimating daily milk yield than ACF. The MCF have biological and statistical challenges. Systematic biases occurred when ACF or MCF were computed on discretized milking interval classes, leading to accuracy loss. An exponential regression model was proposed as an alternative model for estimating daily milk yields, which improved the accuracy. Characterization of ACF and MCF showed how they improved the accuracy compared with doubling AM or PM yield as the daily milk yield. All the methods performed similarly with equal AM and PM milkings. The methods were explicitly described to estimate daily milk yield in AM and PM milking plans. Still, the principles generally apply to cows milked more than 2 times a day and apply similarly to the estimation of daily fat and protein yields with some necessary modifications.

## Highlights

• ACF and MCF are demonstrated and their intrinsic relationships are revealed.
• ACF and MCF improve the accuracy compared with doubling AM or PM milk yield.
• Interpretations of MCF are given, and biological and statistical challenges are discussed.
• Systematic biases arising from discretized milking interval classes when computing ACF and MCF are illustrated.
• The exponential regression model has the smallest biases and the highest accuracies for estimating daily milk yields.

## Graphical Abstract

**Figure:**
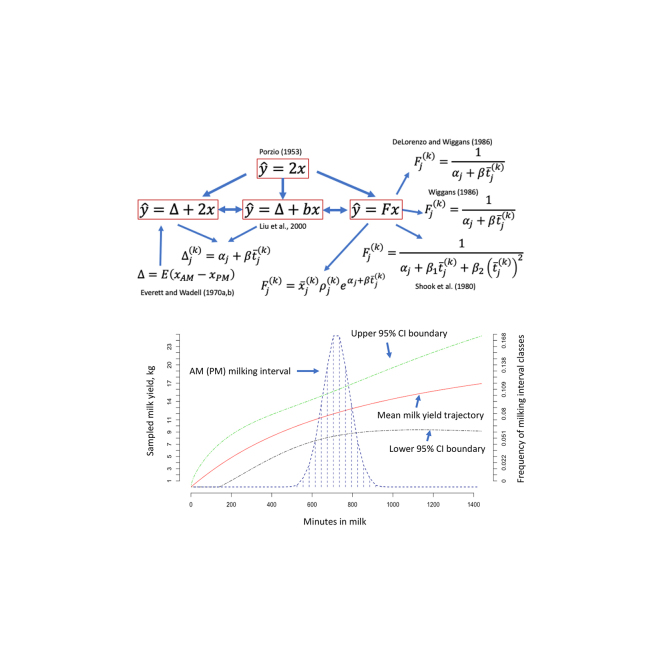
**Summary:** Cows are typically milked 2 or more times on a test-day, but not all those milkings are measured. Statistical methods have been proposed to estimate daily yields, centering on various yield correction factors in 2 broad categories: additive correction factors (ACF) and multiplicative correction factors (MCF). This research note presented a technical review of statistical models for estimating daily milk yields concerning their statistical interpretations, mode assumptions, and challenges. An exponential regression model was proposed as an alternative tool for estimating daily milk yields. The features of ACF and MCF are illustrated using simulation datasets, and their performance was evaluated by 10-fold cross-validations. The methods were described explicitly for estimating daily milk yield in morning (AM) and evening (PM) milking plans, but the principles are generally applicable to cows milked more than 2 times a day, and apply to estimating daily fat and protein yields, with some necessary modifications.

Accurate milking data are essential for herd management and genetic improvement in dairy cattle. Cows are typically milked 2 or more times on a test-day, but not all these milkings are sampled and weighed. This practice started to supplement the standard supervised twice-daily monthly testing scheme in the 1960s, motivated by reducing the visits by a national DHIA supervisor and lowering the costs to dairymen (Putnam and Gilmore, 1968). The initial AM-PM milking plan alternately sampled the morning (**AM**) or evening (**PM**) milking on a test-day throughout the lactation. Daily yield (milk, fat, and protein) was estimated by 2 times the yield from single milkings on each test-day, assuming equal AM and PM milking intervals (Porzio, 1953). Let *x_ij_* be a known AM or PM yield for animal *i* on a test-day, where *j* = 1 for AM milking or *j* = 2 for PM milking. Then, the total test-day yield is estimated by

[1] $${\hat{y}}_{ij}=2x_{ij}.$$

Various methods have been proposed afterward, mainly to deal with varied milking intervals (see graphical abstract). The landmark developments date to the 1980s and 1990s, focusing on yield correction factors in 2 broad categories: additive correction factors (**ACF**) and multiplicative correction factors (**MCF**). In AM-PM plans, ACF provide additive adjustments to 2 times AM or PM milk yield as the estimated daily yield, computed specifically for each milking interval class (**MICL**), say *k*. That is,

[2] $${\hat{y}}_{ijk}=\Delta_{jk}+2x_{ijk}.$$

Here, Δ*_jk_* represents an ACF for milking interval *k* of milking *j*, and ${\hat{y}}_{ijk}$ is the estimated daily yield for cow *i*.

By noting that a daily milk yield equals the sum of the AM and PM milk yield, we obtain the following 2 equations given AM and PM milk yield:

[3] $$\Delta_{2k}=x_{i1k}-x_{i2k},$$

[4] $$\Delta_{1k}=x_{i2k}-x_{i1k}.$$

Thus, ACF are evaluated by the population averages of the differences between the AM and PM milk yield, coupled with other categorical variables (Everett and Wadell, 1970).

By adding equation [3] to equation [4], it shows that the sum of AM and PM ACF specific to each MICL equals zero:

[5] $$\Delta_{1k}+\Delta_{2k}=0.$$

A general form of an equivalent ACF model with daily yield as the response variable is the following:

[6] $$y_{ijk}=f\left(\theta \right)+bx_{ijk}+\varepsilon_{ijk},$$

where *f*(*θ*) is a function with discrete variables, b≡2 is the regression coefficient for AM or PM yield, and *ε_ijk_* is a residual. Then, ACF are evaluated locally as the expected (*E*) values of *f*(*θ*) for each MICL:

[7] $$\Delta_{jk}=E\left[f\left(\theta ;j,k\right)\right].$$

Similarly, a linear regression (**LR**) model can be implemented as an ACF model in which *f*(*θ*) is a linear function involving milking interval and DIM, and the regression coefficient for AM or PM yield is estimated from the data. Let *t_ij_* be a milking interval time and *d_ij_* is the DIM for the test date when *x_ij_* is sampled and weighed; all are pertaining to milking *j*. Then, the LR model involving milking interval time and DIM alone is the following:

[8] $$y_{ij}=\alpha_{j}+\beta t_{ij}+\gamma \left(d_{ij}-d_{0}\right)+bx_{ij}+{*}_{ij}.$$

Here, *α_j_* is an overall mean specific to milking *j*, and *β, γ*, and *b* are common regression coefficients for milking interval, DIM, and single milking (AM or PM) yield, respectively. Note that LR models can be defined with varying complexity by adding or dropping regression variables as appropriate (Liu et al., 2000).

As a typical LR approach, daily yield is estimated directly given the estimated model parameters in [8]. Alternatively, ACF can be obtained by evaluating the expected values of *f*(*θ*) for discretized MICL locally. Assume that *E*(*d_ij_* − *d*_0_) = 0. Let there be *k* = 1, …, *K* classes for AM (or PM) milking. Then, ACF are computed for 2 × *K* classes.

[9] $$\Delta_{j}^{\left(k\right)}=E\left(\alpha_{j}+\beta t_{ij}^{\left(k\right)}\right)=\alpha_{j}+\beta {\bar{t}}_{j}^{\left(k\right)}.$$

The above holds assuming $E\left(t_{ij}^{\left(k\right)}-{\bar{t}}_{j}^{\left(k\right)}\right)$ = $E\left(t_{ij}^{\left(k\right)}\right)-{\bar{t}}_{j}^{\left(k\right)}=0,$ where $E\left({\bar{t}}_{j}^{\left(k\right)}\right)={\bar{t}}_{j}^{\left(k\right)}$ is a midpoint of each MICL. Throughout this paper, we use a superscript *k* to indicate a discretized MICL because it is not a variable index in the data model. In contrast, a subscript index *k* is reserved for an index for a categorical variable in the data model. Then, the daily yield is given by the sum of $\hat{b}x_{ij}$ and the ACF, $\Delta_{j}^{\left(k\right)},$ explicitly computed for the *k*th MICL. That is,

[10] $${\hat{y}}_{ij}={\hat{\Delta }}_{j}^{\left(k\right)}+\hat{b}x_{ij}.$$

Within each MICL, the following holds in a joint analysis using AM and PM milking records, assuming a common regression coefficient for AM or PM yield.

[11] $$\Delta_{1}^{\left(k\right)}+\Delta_{2}^{\left(k\right)}=\left(2-b\right){\bar{y}}^{\left(k\right)},$$

where ${\bar{y}}^{\left(k\right)}$ is the average daily milking yield for all the cows in MICL *k*. Hence, if we force *b* = 2, the above relationship [11] is reduced to [5].

Multiplicative correction factors, also referred to as ratio factors, are ratios of daily yield to yield from single milkings, computed for various MICL (e.g., Shook et al., 1980; DeLorenzo and Wiggans, 1986; Wiggans, 1986). Denote AMP and PMP for bulk AM and PM yield, respectively, such that AMP + PMP gives the test-day yield. Then, the AM and PM MCF, denoted by *F*_1_ and *F*_2_, respectively, are defined as follows (Shook et al., 1980):

[12] $$F_{1}=\frac{AMP+PMP}{AMP},$$

[13] $$F_{2}=\frac{AMP+PMP}{PMP}.$$

Confined to MICL *k*, we show the following relationship holds based on equations [12] and [13]:

[14] $$F_{1k}^{-1}+F_{2k}^{-1}=1.$$

The above brings convenience to computing. For example, given the computed PM MCF (*F*_2_*_k_*), AM MCF can be obtained indirectly as follows:

[15] $$F_{1k}={\left(1-F_{2k}^{-1}\right)}^{-1}=\frac{F_{2k}}{F_{2k}-1}.$$

Shook et al. (1980) utilized a quadratic regression of the PM portion of daily yield on milking interval time to obtain smoothed estimates of MCF and dealt with MICL having no or insufficient milking records.

DeLorenzo and Wiggans (1986) proposed an LR model without intercept to derive MCF for cows milked twice a day, as follows, assuming heterogeneous means and variances and fitted separate LR models for different MICL:

[16] $$y_{ijk}=F_{jk}x_{ijk}+\varepsilon_{ijk}.$$

The above regression coefficient *F_jk_* coincides by definition with the MCF specific to each MICL (Shook et al., 1980), assuming $E\left(\varepsilon_{ijk}\right)$ = 0, because MCF for MICL k is also computed by

[17] $$F_{jk}=\frac{E\left(y_{ijk}\right)}{E\left(x_{ijk}\right)}==\frac{\frac{1}{n}\sum_{i=1}^{n}\;y_{ijk}}{\frac{1}{n}\sum_{i=1}^{n}\;x_{ijk}}=\frac{\sum_{i=1}^{n}\;y_{ijk}}{\sum_{i=1}^{n}\;x_{ijk}}.$$

Here, $\sum_{i=1}^{n}\;x_{ijk}$ corresponds to AMP*_k_* or PMP*_k_* and $\sum_{i=1}^{n}\;y_{ijk}$ corresponds to AMP*_k_* + PMP*_k_*. DeLorenzo and Wiggans (1986) proposed linear smoothing by regressing the reciprocals of computed AM or PM factors on milking interval time to obtain smoothed MCF.

A covariate for DIM can also be included to account for the variation of the lactation curve (DeLorenzo and Wiggans, 1986).

Wiggans (1986) proposed deriving yield factors for cows milked 3 times a day through regressing the AM or PM proportion of daily yield (*x_ij_*/*y_ij_*) on milk interval (*t_ij_*), as follows:

[18] $$\frac{x_{ij}}{y_{ij}}=\alpha_{j}+\beta t_{ij}+\varepsilon_{ij}.$$

By taking the expected value on both sides of equation [18], and letting *E*(*ε_ij_*) = 0, we have

[19] $$E\left(\frac{x_{ij}}{y_{ij}}\right)=\alpha_{j}+\beta E\left(t_{ij}\right).$$

Here, $E\left(t_{ij}\right)={\bar{t}}_{j}^{\left(k\right)}$ is evaluated locally as the midpoint of each MICL for milking *j*. Then, MCF are obtained as

[20] $$F_{j}^{\left(k\right)}=E\left(\frac{y_{ij}^{\left(k\right)}}{x_{ij}^{\left(k\right)}}\right)={\left(E\left(\frac{x_{ij}^{\left(k\right)}}{y_{ij}^{\left(k\right)}}\right)\right)}^{-1}=\frac{1}{{\hat{\alpha }}_{j}+\hat{\beta }{\bar{t}}_{j}^{\left(k\right)}}.$$

The statistical interpretations of MCF vary slightly. First, according to DeLorenzo and Wiggans (1986), an MCF is a regression coefficient specific to each MICL, defined in [16]. Second, an MCF is a ratio of the expected value of daily yield to the expected value of the yield from single milkings, defined in [17] and computed for each MICL (Shook et al., 1980; DeLorenzo and Wiggans, 1986). Third, an MCF is the expected value of the ratio of daily yield to a single milking yield according to the Wiggans (1986) model, defined in [20]. Note that the third interpretation can also be derived from the regression smoothing models by Shook et al. (1980) and DeLorenzo and Wiggans (1986), because they fitted the same or similar ratio variable in the linear or quadratic smoothing models. The 3 forms of MCF represent different strategies or formulations for estimating ratios of daily yield to yield from single milkings. Nevertheless, they correspond to each other approximately. For example, the form in [20] approximately agrees with [17] if we apply the first-order Taylor approximation to [20]. That is,

[21] $$E\left(\frac{y_{ij}}{x_{ij}}\right)\approx \frac{E\left(y_{ij}\right)}{E\left(x_{ij}\right)}.$$

Multiplicative correction factor models are statistically challenged by the well-known “ratio problem” because they have a ratio variable (e.g., AM or PM proportion of daily yield) as the dependent variable in the data density (Wiggans, 1986) or the smoothing functions (Shook et al., 1980; DeLorenzo and Wiggans, 1986). The consequences included possible biases in 2 aspects: omitted variable bias and measurement error bias (Lien et al., 2017). The former happens because the main model effects are missing if the model is re-arranged by multiplying the denominator variable to both sides of the equation. The latter occurs when there are measurement errors with the denominator variable of the response.

Here, we propose an alternative model by taking the logarithm of daily to single milking (say AM or PM) yield ratio as a response variable. That is,

[23] $$\log\left(y_{ij}/x_{ij}\right)=\alpha +\beta t_{ij}+{ɛ}_{ij},$$

where (*y_ij_*/*x_ij_*) is a ratio of daily yield to single milking (say AM or PM) yield. With some re-arrangements, equation [22] becomes

[23] $$log\left(y_{ij}\right)=\alpha_{j}+\beta t_{ij}+blog\left(x_{ij}\right)+\varepsilon_{ij}.$$

Here, log(*y_ij_*) is the response variable, log(*x_ij_*) and *t_ij_* are the dependent variables (i.e., main effects), and *b* = 1 is a constant regression coefficient for log(*x_ij_*). In the present study, however, we relax the restriction for *b* = 1 in [23] and estimate it from the data. Then, taking the exponential on both sides of equation [23] gives

[24] $$y_{ij}=x_{ij}^{b}\;e^{\left(\alpha_{j}+\beta t_{ij}+\varepsilon_{ij}\right)}.$$

The above is recognized as an exponential regression model. The model parameters can be conveniently estimated with data fitted on the linear logarithm equation [23]. Daily yield is calculated given the model parameter estimates $\left(\hat{b},{\hat{\alpha }}_{j},\hat{\beta }\right),$ and observed partial (AM or PM) yield and milking interval time.

To derive MCF, we first take expected values on both sides of the equation [24]. Then, we applied the second order Taylor approximation by noting that *E*[log(*z*)] ≈ log[*E*(*z*)] − [*V*(*z*)/2*E*(*z*)^2^], where *z* is a random variable. Hence, we have

[25] $$\begin{array}{l} log\left[E\left(y_{i}\right)\right]=\alpha_{j}+\beta \left[E\left(t_{ij}\right)\right]+blog\left[E\left(x_{ij}\right)\right]\\ +\left(\frac{V\left(y_{ij}\right)}{2E{\left(y_{ij}\right)}^{2}}-b\frac{V\left(x_{ij}\right)}{2E{\left(x_{ij}\right)}^{2}}\right). \end{array}$$

Next, taking the exponential on both sides of equation [25], with some re-arrangements, gives

[26] $$E\left(y_{ij}\right)=\rho E{\left(x_{ij}\right)}^{b}e^{\left\{\alpha_{j}+\beta E\left(t_{ij}\right)\right\}},$$

where $\rho =e^{\frac{1}{2}\left(V\left(y_{ij}\right)E{\left(y_{ij}\right)}^{-2}-bV\left(x_{ij}\right)E{\left(x_{ij}\right)}^{-2}\right)}.$

The MCF are derived by evaluating the expected values of [26] locally for each MICL, say *k*, and dividing both sides of the equation by $E\left(x_{ij}^{\left(k\right)}\right).$

That is,

[27] $$F_{j}^{\left(k\right)}=\frac{E\left(y_{ij}^{\left(k\right)}\right)}{E\left(x_{ij}^{\left(k\right)}\right)}=\rho_{j}^{\left(k\right)}E{\left(x_{ij}^{\left(k\right)}\right)}^{b-1}e^{\alpha_{j}+\beta {\bar{t}}_{j}^{\left(k\right)}},$$

where $\rho_{j}^{\left(k\right)}=e^{\frac{1}{2}\left(V\left(y_{ij}^{\left(k\right)}\right)E{\left(y_{ij}^{\left(k\right)}\right)}^{-2}-bV\left(x_{ij}^{\left(k\right)}\right)E{\left(x_{ij}^{\left(k\right)}\right)}^{-2}\right)},$ and $E\left(y_{ij}^{\left(k\right)}\right)={\bar{y}}_{j}^{\left(k\right)}$ and $E\left(x_{ij}^{\left(k\right)}\right)={\bar{x}}_{j}^{\left(k\right)}$ are the corresponding means for daily yield and AM (or PM) yield, respectively.

A simulation study was conducted. Daily milk yields were simulated based on a modified Michaelis-Menten function (Klopcic et al., 2013). Daily milk yield curves were simulated for 3,000 cows (see graphical abstract), where the values for *y*_720_ and *k* were simulated from truncated normal (**TN**) distributions: *y*_720_ ~ TN (12, 2) and *k* ~ TN (0.8, 0.1). The AM milking intervals were simulated following a TN distribution with a mean equaling 12 h and a standard deviation of 1.12 h. PM milking intervals for the same cows were 24 h minus the AM milking intervals. Approximately 98.6% of the cows had AM (PM) milking intervals between 9 and 15 h (see graphical abstract). Deviations due to DIM and other system variables were all ignored.

The performance of each model was evaluated based on mean squared errors (**MSE**) and accuracies of estimated daily milk yields obtained from 10-fold cross-validations, each replicated M = 30 times. The R^2^ accuracy (Liu et al., 2000) was computed per cross-validation replicate and per individual animal except that the MSE was obtained from the testing sets. Hence, it can be reviewed as predictive R^2^ accuracy. To further infer the origin of errors, MSE was decomposed into variance and squared bias, computed as an average of all animals per cross-validation replicate or as the average for each animal across the 30 replicates.

The variances of estimated daily milk yields were all close to zero for these methods (Table 1), suggesting that they all had high precision for the estimates. Overall, ACF and MCF models considerably outperformed M0 (double AM or PM yields) regarding biases and accuracies (Table 1). The 2 ACF models, M1 and M2B, had larger MSE and lower R^2^ accuracies than M2A. For the MCF models, M6A and M6B (Wiggans, 1986) performed slightly better than M4 (Shook et al., 1980) and M5 (DeLorenzo and Wiggans, 1986). The latter 2 models, M4 and M5, performed similarly. Model M6B estimated daily milk yield based on MCF (Wiggans, 1986). The 2 exponential function models, M7A and M7B, had the smallest squared bias (and MSE) and the largest accuracies in the methods. The ranges of accuracies between cross-validation replicates were very narrow. The accuracies evaluated per animals were slightly higher than those assessed per cross-validation replicates, and the ranges of accuracies in the latter case were drastically larger. The accuracy ranges were between 0.356 and 1.00 (M7B) and between 0.363 and 1.00 (M7A), respectively, based on the exponential regression models, whereas the accuracy ranges were larger for other methods. M0 had the lowest accuracy (0.730) and the most extensive range (0.153–0.788) per animal (Table 1).

**Table 1:** Variance, squared bias (bias^2^), and mean squared error (MSE) of estimated daily milk yields using different models and strategies

Model^1^	Per cross-validation replicate				Per animal
	Variance	Bias^2^	MSE	Mean accuracy (range)	Mean accuracy (range)
M0	<0.001	5.940	5.940	0.714 (0.7143–0.7143)	0.730 (0.153–0.788)
M1	<0.001	0.486	0.486	0.968 (0.9684–0.9688)	0.976 (0.319–1.00)
M2A	<0.001	0.448	0.448	0.971 (0.9708–0.9709)	0.978 (0.320–1.00)
M2B	<0.001	0.480	0.480	0.968 (0.9678–0.9679)	0.976 (0.316–1.00)
M3A	<0.001	0.435	0.435	0.972 (0.9724–0.9725)	0.979 (0.331–1.00)
M3B	<0.001	0.465	0.465	0.970 (0.9695–0.9696)	0.977 (0.327–1.00)
M4	<0.001	0.422	0.422	0.972 (0.9715–0.9745)	0.978 (0.333–1.00)
M5	<0.001	0.420	0.421	0.972 (0.9716–0.9719)	0.978 (0.341–1.00)
M6A	<0.001	0.386	0.386	0.975 (0.9751–0.9752)	0.980 (0.352–1.00)
M6B	<0.001	0.417	0.417	0.973 (0.9729–0.9730)	0.978 (0.347–1.00)
M7A	<0.001	0.376	0.376	0.976 (0.9763–0.9765)	0.982 (0.363–1.00)
M7B	<0.001	0.385	0.385	0.975 (0.9750–0.9752)	0.980 (0.356–1.00)

1 Model: M1 = doubling AM or PM milk yield; M2A = ACF model implemented as a factorial model with categorical milking interval classes (MICL); M2B = ACF model with categorial MICL; M3A = linear regression with continuous MIT; M3B = linear regression implemented as an ACF model; M4 = MCF model according to Shook et al. (1980); M5 = MCF model according to DeLorenzo and Wiggans (1986); M6A = MCF model implemented as linear regression according to Wiggans (1986); M6B = MCF model according to Wiggans (1986); M7A = exponential regression model; M7B = MCF model based on exponential regression. All ACF and MCF were evaluated on MIT or MICL only.

Estimated model parameters for 4 models were obtained in one cross-validation replicate and shown in Table 2. The correlation coefficients between actual and estimated daily milk yields were high for all the models. Yet, the fitted linear regressions between actual and estimated daily milk yields varied considerably between these models. The ACF model (M1) had larger intercepts than the MCF model. The LR model with continuous milking interval (M2A) had a significantly smaller intercept. The exponential regression model, M7A, had the smallest intercept, and the regression coefficient was close to 1. Therefore, the ACF model M1 had the largest biases and the worst accuracies. The exponential model M7A had the least biases and the greatest accuracies. Hence, correlations are not an appropriate measure of accuracy for estimating daily milk yields because biases are not considered.

**Table 2:** Estimated model parameters, linear regression fits, and corrections between actual (*y*) and estimated $\left(\hat{y}\right)$ daily milk yields obtained from 4 statistical models^1^, ^2^, ^3^, ^4^

Statistical model	Model parameter					
	*α_AM_*	*α_PM_*	*β*	*b*	Linear regression fit	Correlation
M2A						
	14.17	14.19	−1.182	2.0	AM: $y=0.822+0.966\hat{y}$	0.985
	(0.095)	(0.095)	(0.008)	—	PM: $y=0.580+0.976\hat{y}$	0.985
M3A						
	14.46	14.48	−1.147	1.942	AM: $y=0.123+0.995\hat{y}$	0.986
	(0.096)	(0.096)	(0.008)	(0.004)	PM: $y=-0.126+1.005\hat{y}$	0.985
M6A						
	0.208	0.208	0.024	—	AM: $y=0.684+0.972\hat{y}$	0.986
	(0.002)	(0.002)	(<0.001)	—	PM: $y=0.577+0.976\hat{y}$	0.986
M7A						
	1.324	1.324	−0.048	0.977	AM: $y=0.102+0.996\hat{y}$	0.988
	(0.005)	(0.005)	(<0.001)	(0.002)	PM: $y=-0.009+1.001\hat{y}$	0.987

1 M2A, M3A, M6A, M7A: see model specifications in Table 1.

2 Each model assumed heterogeneous intercepts for AM and PM milking (*α*_AM_ and *α*_PM_), respectively, and a common regression coefficient (*β*) for milking interval; *b* = regression coefficient of single milking (AM or PM) yield.

3 Numbers in parentheses are standard deviations of estimated model parameters.

4 — = not applicable.

The impact of discretizing milking intervals on estimating daily milk yields was evaluated through 4 pairs of models. Each pair had the same model settings, except daily milk yields were calculated using different strategies. The models labeled A (M2A, M3A, M6A, and M7A) estimated daily milk yields directly based on estimated model parameters. Instead, the models labeled B (M2B, M3B, M6B, and M7B) first computed ACF or MCF for discretized MICL. Then, daily milk yields were estimated based on the computed ACF or MCF per discretized MICL. The models in group A consistently had smaller MSE and greater predictive R^2^ accuracies than their counterparts in group B. We thus concluded that discretizing milking interval time led to increased biases and, therefore, loss of accuracies in estimated daily milk yields. How systematic biases arose from discretizing milking interval is analytically shown below. Consider the LR model M3A. Given the model parameters, it estimated daily milk yield as follows:

[28] $${\hat{y}}_{ij}={\hat{\alpha }}_{j}+\hat{\beta }t_{ij}+\hat{b}x_{ij}=\left({\hat{\alpha }}_{j}+\hat{\beta }{\bar{t}}_{j}^{\left(k\right)}\right)+\hat{\beta }\left(t_{ij}-{\bar{t}}_{j}^{\left(k\right)}\right)+\hat{b}x_{ij}.$$

The above estimated daily milk yield consisted of 3 components. In contrast, model M3B estimated daily milk yield by the first and the third components in [28]:

[29] $${\hat{y}}_{ij}=\left({\hat{\alpha }}_{j}+\hat{\beta }{\bar{t}}_{j}^{\left(k\right)}\right)+\hat{b}x_{ij},$$

where ${\hat{\alpha }}_{j}+\hat{\beta }{\bar{t}}_{j}^{\left(k\right)}$ corresponds to an ACF. Hence, the second term on the right-hand side of [28], $\hat{\beta }\left(t_{ij}-{\bar{t}}_{j}^{\left(k\right)}\right),$ was ignored, which represented a systematic bias. Similar situations were held for the models M2A and M2B. For the exponential model M7B, the bias due to discretizing interval milking was quantified by $e^{\hat{\beta }\left(t_{ij}-{\bar{t}}_{j}^{\left(k\right)}\right)}.$

Biases due to discretizing milking interval also existed with the model M6B (Wiggans, 1986). Nevertheless, the biases from discretizing milking intervals were relatively small in the present study.

Additive and multiplicative correction factors were characterized and compared in Figure 1. The ACF computed from the 2 ACF models, M1 and M2B, were comparable, except that ACF from model M2B were smoothed. But they did not agree with ACF computed from the LR model M3B. The average difference of ACF per MICL between M2B and M3B was 1.402/2 = 0.701. Numerically, we approximated the results by deriving a similar average difference of ACF between these 2 models:

$$\begin{array}{l} {\bar{\Delta }}_{M3B}-{\bar{\Delta }}_{M2B}=\frac{1}{K}\sum_{k=1}^{K}\;\left({\bar{\Delta }}_{M3B}^{\left(k\right)}-\Delta_{M2B}^{\left(k\right)}\right)=\\ \left(2-b\right)\left(\frac{1}{2K}\sum_{j=1}^{2}\;\sum_{k=1}^{K}\;{\bar{x}}_{j}^{\left(k\right)}\right)\approx 0.699, \end{array}$$

where *b* = 1.942, and $\frac{1}{2K}\sum_{j=1}^{2}\;\sum_{k=1}^{K}\;{\bar{x}}_{j}^{\left(k\right)}$ = 12.05. The sums of AM and PM ACF within MICL were all close to zero for the ACF model. For the LR model, however, the sums of AM and PM ACF within MICL was approximately 1.402. Analytically, the sum was estimated to be $\left(2-\hat{b}\right)\left(\frac{1}{2K}\sum_{j=1}^{2}\;\sum_{k=1}^{K}\;{\bar{y}}_{j}^{\left(k\right)}\right)$ = 1.398 according to [11], where $\frac{1}{2K}\sum_{j=1}^{2}\;\sum_{k=1}^{K}\;{\bar{y}}_{j}^{\left(k\right)}$ = 24.10.

**Figure 1:**
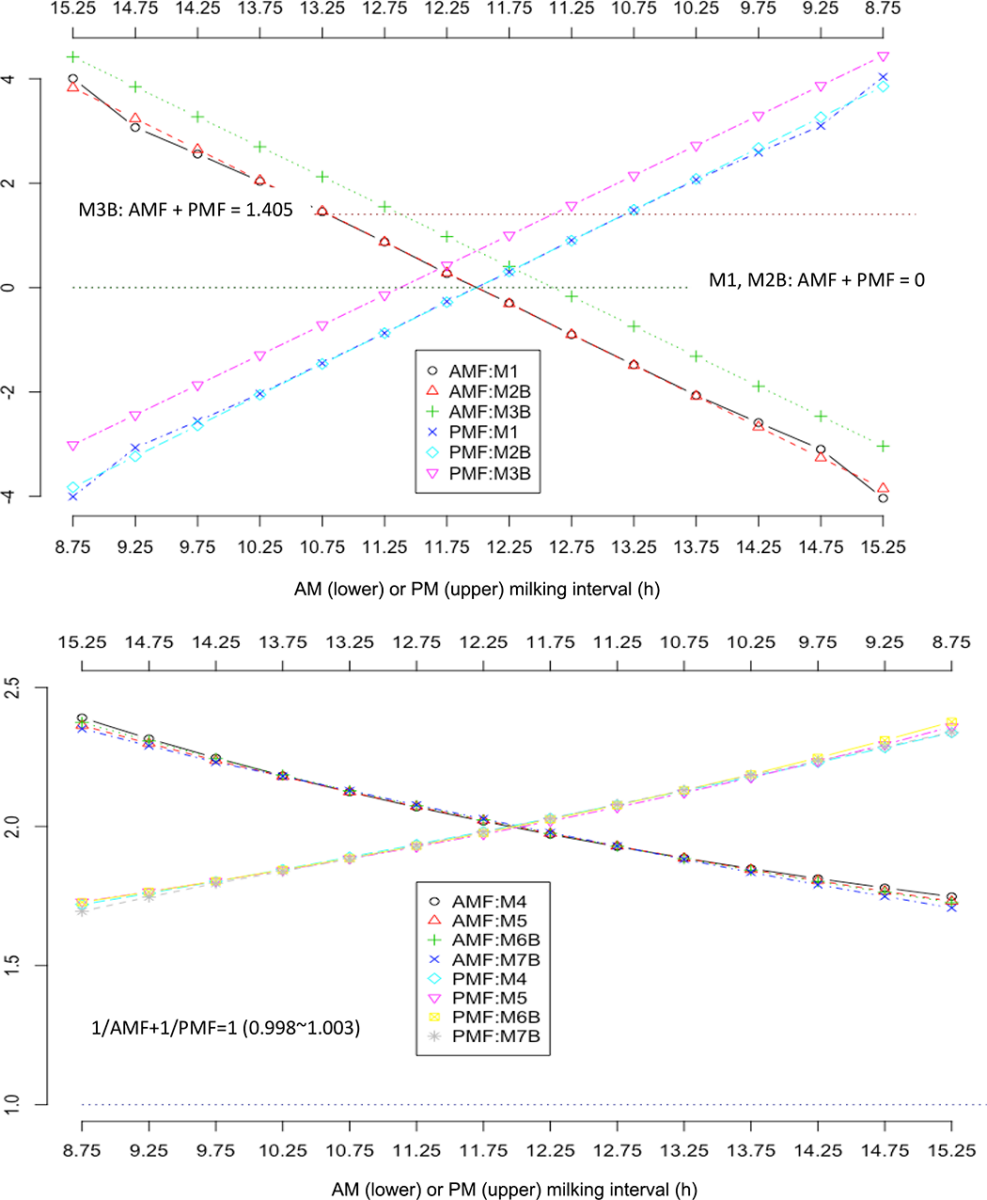
Comparing additive correction factors (upper) and multiplicative correction factors (lower) obtained using different methods. M1, M2B, M3B, M4, M5, M6B, M7B, M8B: see model specifications in Table 1. AMF = morning correction factors; PMF = evening correction factors.

Multiplicative correction factors were computed and compared between 4 models: M4 (Shook et al., 1980), M5 (DeLorenzo and Wiggans, 1986), M6B (Wiggans, 1986), and M7B. Overall, MCF computed from different models are highly comparable for milking intervals between 9 and 14 h, but they showed relatively larger differences out of this range (Figure 1). All the computed MCF provided estimates of the ratios of daily yield to yields from single milkings, though their precise statistical interpretations varied. ACF from the model M1 and M2B were zero when AM and PM milking intervals were both approximately 12 h, meaning that close to zero adjustments were added to 2 times AM or PM milk yield as the estimated daily milk yields. MCF were all close to 2.0 with approximately 12–12 h equal AM and PM milking intervals. The MCF became smaller or larger than 2.0 as AM or PM milking interval departure from 12 to 12 h. Hence, doubling AM or PM milk yield gave an approximate estimate of daily milk yield with equal AM and PM milking intervals. Still, it was subject to large errors when AM or PM milking interval deviated from 12 h.

By noting *e* ≈ 2.718, we show that the exponential function is analogous to an exponential growth function:

[30] $$y=x^{b}{\left(1+1.718\right)}^{t^{*}},$$

where *y*_0_ = *x^b^* is the initial value, *r* = 1.718 is the rate of change, tuned by a time function as a linear function of milking interval and DIM. Thus, the proposed model (M7A and M7B) postulated exponential growth dynamics between daily milk yield and milking interval time, given known AM or PM yield as the initial value.

Finally, we reviewed and evaluated ACF and MCF models using simulation data, yet to be verified by actual milking data. In a continuing effort, large-scaled high-resolution milking data are being collected for follow-up studies, jointly supported by the US Council on Dairy Cattle Breeding, the USDA Agricultural Genomics and Improvement Laboratories, and the National Dairy Herd Information Association. The methods were explicitly described to estimate daily milk yield in AM and PM milking plans. Still, the principles generally apply to cows milked more than twice daily and apply similarly to the estimation of daily fat and protein yields with some necessary modifications.
